# Complete Genome Sequence of Acinetobacter schindleri SGAir0122 Isolated from Singapore Air

**DOI:** 10.1128/genomeA.00567-18

**Published:** 2018-06-28

**Authors:** Carmon Kee, Ana Carolina M. Junqueira, Akira Uchida, Rikky W. Purbojati, James N. I. Houghton, Caroline Chénard, Anthony Wong, Megan E. Clare, Kavita K. Kushwaha, Deepa Panicker, Alexander Putra, Nicolas E. Gaultier, Balakrishnan N. V. Premkrishnan, Cassie E. Heinle, Vineeth Kodengil Vettath, Daniela I. Drautz-Moses, Stephan C. Schuster

**Affiliations:** a Singapore Centre for Environmental Life Sciences Engineering, Nanyang Technological University, Singapore; b Asian School of the Environment, Nanyang Technological University, Singapore

## Abstract

Acinetobacter schindleri strain SGAir0122 was isolated from tropical air samples collected in Singapore. The prevalence of nosocomial infection caused by this Gram-negative bacterium indicates its clinical significance as an opportunistic human pathogen. Its complete genome consists of one chromosome of 3.105 Mb and a plasmid of 181 kb.

## Abstract (precis)

Acinetobacter schindleri strain SGAir0122 was isolated from tropical air samples collected in Singapore. The prevalence of nosocomial infection caused by this Gram-negative bacterium indicates its clinical significance as an opportunistic human pathogen.

## GENOME ANNOUNCEMENT

Acinetobacter schindleri is a nonmotile, aerobic, Gram-negative bacterium (1). A. schindleri is an opportunistic human pathogen affecting patients in intensive care units and immunocompromised patients (2), contributing to nosocomial infections and outbreaks worldwide (3). This species can be found in a wide range of natural environments, such as in soil and water, as well as in hospitals (1), due to its ability to survive on inert surfaces for a long duration (4). Phenotypic identification of the isolates remains indiscriminative and may lead to underestimation of infections caused by A. schindleri; thus, the complete genome sequences of A. schindleri strains may be of great help in providing molecular markers for identification.

Air samples were collected outdoor in Singapore (1.345°N, 103.679°E) using an Andersen single-stage impactor (SKC, Inc., USA). Strain SGAir0122 was isolated from trypticase soy agar (Becton, Dickinson, USA) incubated at 30˚C after airborne microorganisms were impacted onto it. The isolated strain was cultured in Luria-Bertani broth overnight at room temperature. Prior to DNA extraction, an axenic culture was obtained by streaking. Genomic DNA was extracted using the Wizard genomic DNA purification kit (Promega, USA) according to the manufacturer’s recommended protocol. Whole-genome shotgun libraries were constructed using a SMRTbell template prep kit version 1.0 (Pacific Biosciences, USA), followed by single-molecule real-time (SMRT) sequencing conducted on a PacBio RS II (Pacific Biosciences) sequencer.

The PacBio RS II platform provided 68,481 long reads (208-fold coverage) for the isolate. The complete processing of the assembly included preassembly, *de novo* assembly with the Hierarchical Genome Assembly Process (HGAP) version 3 implemented in the PacBio SMRT Analysis algorithm version 2.3.0 (5), polishing with Quiver (5), and error correction using Pilon version 1.16 (6) to improve accuracy. The consensus assembly generated two contigs, one chromosome with 3,104,554 bp (202-fold coverage), and one plasmid with 180,768 bp (321-fold coverage). The chromosomal contig showed an average G+C content of 42.7%.

The complete genome presented 99.3% marker identity with that of the Acinetobacter genus, as predicted by the phylum-specific automated phylogenomic inference pipeline Phyla_AMPHORA using a minimum confidence of 1.00 (7). Further taxonomical identification was conducted using the average nucleotide identity method with the Microbial Species Identifier (MiSI) (8), which revealed a 97.5% similarity with the available reference genome of A. schindleri CIP 107287 (GenBank accession number KB849571).

Annotation of the genome was performed with the NCBI Prokaryotic Genome Annotation Pipeline (PGAP) version 4.2 (9). The complete genome was predicted to contain 3,206 genes, including 2,850 protein-coding genes, 21 rRNA subunits (5S, 16S, and 23S), 86 tRNAs, and 4 noncoding RNAs, as well as 245 pseudogenes. Functional annotation using the Rapid Annotations using Subsystems Technology (RAST) server (10–12) classified 96 genes as being associated with virulence and defense, indicating a moderate virulence. There were 120 genes found to have a role in stress response and 20 genes found to be involved in secondary metabolism. This could confer the organism’s advantages to outcompete other bacteria with secondary metabolites and contribute to its long-term survival on inert surfaces.

### Accession number(s).

The complete genome sequence of A. schindleri strain SGAir0122, including the plasmid pSGAir0122, has been deposited in DDBJ/EMBL/GenBank under the accession numbers CP025618 (chromosome) and CP025619 (plasmid pSGAir0122).
